# Supplementary material for: Ultrasound Diagnostic and Physiotherapy Approach for a Patient with Parsonage–Turner Syndrome—A Case Report
Source: Sensors (Basel). 2023 Jan 2;23(1):501. doi: 10.3390/s23010501 (PMC9824188; doi:10.3390/s23010501)
Supplement: Supplementary file 1 [file sensors-23-00501-s001.zip › sensors-2080577-supplementary.pdf]

Supplement material legends

Table S1. Care checklist – case study

Topic	Item	Checklist item description	Reported on Line
**Title**	1	The diagnosis or intervention of primary focus followed by the words “case report”.	2-3
**Key Words**	2	2 to 5 key words that identify diagnoses or interventions in this case report, including “case report”.	29-30
**Abstract (no references)**	3a	Introduction: What is unique about this case and what does it add to the scientific literature?	15-16
	3b	Main symptoms and/or important clinical findings.	16-17, 22-23
	3c	The main diagnoses, therapeutic interventions, and outcomes.	17-20, 21-22, 22-24
	3d	Conclusion—What is the main “take-away” lesson(s) from this case?	24-28
**Introduction**	4	One or two paragraphs summarising why this case is unique (may include references).	56-63, 78-79
**Patient information**	5a	De-identified patient specific information.	83-86
	5b	Primary concerns and symptoms of the patient.	97-98 (Table 1)
	5c	Medical, family, and psycho-social history including relevant genetic information.	85
	5d	Relevant past interventions with outcomes.	No
**Clinical Findings**	6	Describe significant physical examination (PE) and important clinical findings.	97-98 (Table 1)
**Timeline**	7	Historical and current information from this episode of care organized as a timeline.	97-98 (Table 1)
**Diagnostic Assessment**	8a	Diagnostic testing (such as PE, laboratory testing, imaging, surveys).	97-98 (Table 1)
	8b	Diagnostic challenges (such as access to testing, financial, or cultural).	No
	8c	Diagnosis (including other diagnoses considered).	97-98 (Table 1)
	8d	Prognosis (such as staging in oncology) where applicable.	No
**Therapeutic intervention**	9a	Types of therapeutic intervention (such as pharmacologic, surgical, preventive, self-care).	97-98 (Table 1), 151-189

	9b	Administration of therapeutic intervention (such as dosage, strength, duration).	186-189
	9c	Changes in therapeutic intervention (with rationale).	No
**Follow-up and Outcomes**	10a	Clinician and patient-assessed outcomes (if available).	191-211
	10b	Important follow-up diagnostic and other test results.	208-211
	10c	Intervention adherence and tolerability. (How was this assessed?)	194-196
	10d	Adverse and unanticipated events.	No
**Discussion**	11a	A scientific discussion of the strengths AND limitations associated with this case report.	301-303
	11b	Discussion of the relevant medical literature with references.	270-296
	11c	The scientific rationale for any conclusions (including assessment of possible causes).	276-283
	11d	The primary "take-away" lessons of this case report (without references) in a one paragraph conclusion.	311-321
**Patient Perspective**	12	The patient should share their perspective in one to two paragraphs on the treatment(s) they received.	208-211
**Informed Consent**	13	Did the patient give informed consent? Please provide if requested.	Yes

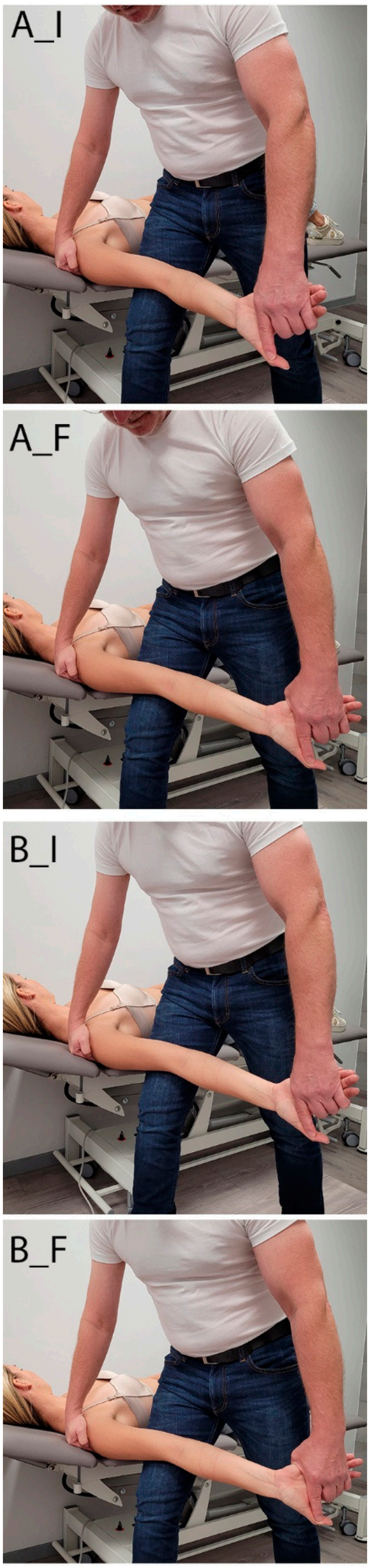

Figure S1. A- Proximal median nerve neurodynamic technique; **B**- Distal median nerve neurodynamic technique; **I** – initial position, **F** – final position.

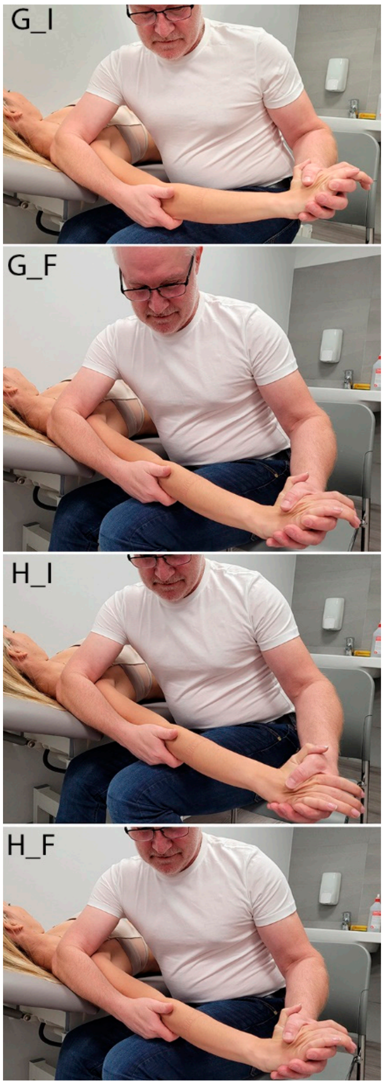

Figure S2. G- Proximal ulnar nerve neurodynamic technique; **H**- Distal ulnar nerve neurodynamic technique; **I** – initial position, **F** – final position.

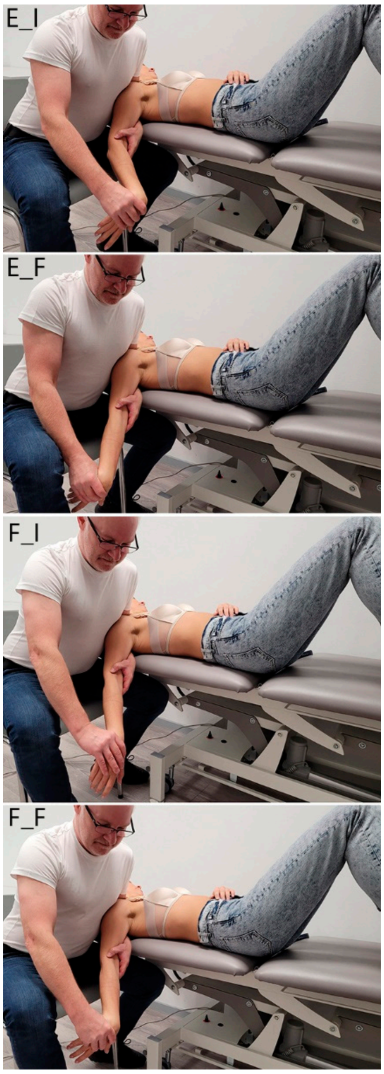

Figure S3. E- Proximal radial nerve neurodynamic technique; **F**- Distal radial nerve neurodynamic technique; **I** – initial position, **F** – final position.

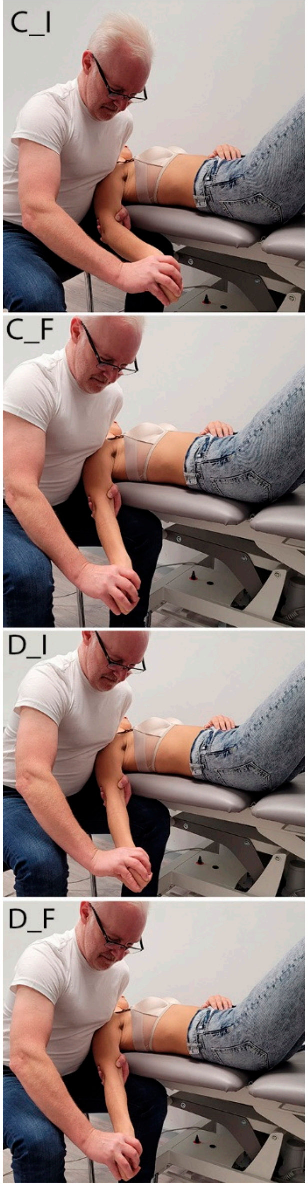

Figure S4. **C**- Proximal musculocutaneus nerve neurodynamic technique; **D**- Distal musculocutaneus nerve neurodynamic technique; **I** – initial position, **F** – final position.
